# Immunoscore and beyond: evolution and clinical integration of immune contexture-based biomarkers across solid tumours

**DOI:** 10.3389/fimmu.2026.1832715

**Published:** 2026-05-08

**Authors:** Esteban Orenes-Piñero, Silverio Ros-Martínez, José Luis Alonso-Romero, Juan Antonio Ortega-García

**Affiliations:** 1 Department of Biochemistry and Molecular Biology, University of Murcia, Murcia, Spain; 2 Proteomic Unit, Instituto Murciano de Investigaciones Biosanitarias (IMIB) Pascual Parrilla, Murcia, Spain; 3 Department of Oncology, Hospital Clínico Universitario Virgen de la Arrixaca (HCUVA), Murcia, Spain; 4 Paediatric Environmental Health Specialty Unit, Hospital Clínico Universitario Virgen de la Arrixaca (HCUVA), Murcia, Spain

**Keywords:** bladder cancer, gastric cancer, head and neck squamous cell carcinoma, hepatocellular carcinoma, Immunoscore, melanoma, non-small cell lung cancer

## Abstract

The tumour immune microenvironment is increasingly recognised as a critical determinant of cancer progression, prognosis, and therapeutic response, challenging the sufficiency of anatomy-based staging systems such as TNM. The Immunoscore (IS), originally validated in colorectal cancer, provides a standardised, spatially resolved assessment of CD3^+^ and CD8^+^ T cells within the tumour core (TC) and invasive margin (IM). Expanding upon this framework, adapted and modified IS models, as well as computational and imaging-based surrogates, have been investigated across multiple solid malignancies, including non-small-cell lung cancer, hepatocellular carcinoma, head and neck cancers, gastric cancer, melanoma, and bladder cancer. This review synthesises the current evidence, emphasising both the translational potential and methodological heterogeneity of IS-based approaches. High IS or adapted scores generally correlate with improved survival and may refine risk stratification, complementing conventional TNM staging. However, inter-study variability limits comparability and reproducibility. Retrospective designs, single-centre cohorts, limited external validation, and potential overfitting in computational models further constrain the strength of evidence. Biological context dependency, immune-exclusion phenotypes, and the dynamic evolution of the tumour immune landscape complicate interpretation, underscoring that high immune cell density does not universally equate to effective antitumour immunity. Overall, the IS and related immune-contexture classifiers constitute a biologically grounded framework that bridges tumour immunology and clinical oncology. While promising, widespread clinical implementation requires methodological harmonisation, prospective validation, and integration with genomic, systemic, and imaging biomarkers. By highlighting both achievements and current limitations, this review provides a balanced perspective on the potential and challenges of translating immune contexture-based metrics into precision oncology.

## Highlights

- Originally validated in colorectal cancer, the Immunoscore (IS) has demonstrated prognostic performance.
- Parallel advances in digital pathology, radiomics, transcriptomics, and integrative modelling have further propelled the IS from a histopathological descriptor to a multidimensional biomarker.
- The IS and related composite immune classifiers provide prognostic and predictive information in several malignancies.
- The IS constitutes a biologically grounded framework that bridges tumour immunology and clinical oncology, capable of informing prognosis, recurrence risk, and response to systemic therapies, including immunotherapy.

## 1 Introduction

Tumour staging [American Joint Committee on Cancer and the Union for International Cancer Control (AJCC/UICC TNM) TNM classification] summarises data on the extent of the tumour burden (T), the presence of cancer cells in draining and regional lymph nodes (N), and evidence of metastases (M) (1). Importantly, in daily practice and in guidelines, the TNM category is directly linked to treatment strategies, and as such, changes in the TNM staging system have a considerable and direct impact on the cancer care that patients receive (2). Unfortunately, the predictive accuracy of the traditional staging system assumes that disease progression is a tumour cell-autonomous process, but it does not take into consideration the effects of the host immune response (2). The interplay between cancer and immune cells is a major determinant in cancer progression, and the immune system is emerging as a powerful prognostic marker and therapeutic target in oncology (3), with tumour-infiltrating lymphocytes (TILs) amongst the potential biomarkers for cancer prognosis.

TILs are a heterogeneous population comprising mainly T lymphocytes and, to a lesser degree, B lymphocytes and natural killer (NK) cells. Contrary to infiltration of cells responsible for chronic inflammation (macrophages, B lymphocytes, eosinophils, or mast cells), the presence of high numbers of T lymphocytes has been reported to be an indicator of good prognosis in many cancers (4). While T lymphocytes represent the core component of most immune-based classifiers, additional immune populations have recently emerged as relevant contributors to tumour immunity. Increasing evidence indicates that other immune components, particularly B cells (e.g., CD20^+^ populations) and tertiary lymphoid structures (TLSs), also contribute to the prognostic and functional landscape of the tumour microenvironment. TLSs are organised lymphoid aggregates resembling secondary lymphoid organs that support local antigen presentation, B-cell maturation, and T-cell priming. Their presence has been consistently associated with improved survival and enhanced response to immune checkpoint blockade across multiple tumour types, often reflecting a coordinated and productive antitumour immune response (5). Similarly, tumour-infiltrating B cells have been linked to favourable outcomes through antibody production, antigen presentation, and modulation of T-cell activity (6). Nevertheless, these components are not currently incorporated into the canonical Immunoscore (IS), which was intentionally designed as a standardised, reproducible, and T cell-centric metric. While expanding immune profiling to include B cell-related features and TLSs may further refine prognostic models, their integration into routine scoring systems remains limited by methodological heterogeneity and lack of standardisation. As such, they represent a complementary, rather than substitutive, dimension of immune contexture that could be incorporated into next-generation IS-based frameworks.

Tumour progression is not solely dictated by malignant cell-intrinsic features but is profoundly influenced by the surrounding tumour microenvironment (TME), particularly its immune component. Patients with comparable tumour burden and identical TNM stage often experience markedly different clinical outcomes, underscoring the limitations of anatomy-based staging systems in capturing biological heterogeneity. TILs, especially cytotoxic and memory T-cell populations, play a central role in antitumour immunity and have been repeatedly associated with survival and therapeutic response across cancer types.

The IS was developed to translate immune contexture into a standardised, reproducible metric by quantifying CD3^+^ and CD8^+^ T cells within the tumour core (TC) and invasive margin (IM) (1). Originally validated in colorectal cancer, the IS has been shown to be a complement to the TNM traditional cancer staging in several malignancies. Thus, TNM-IS combines traditional TNM cancer staging (Tumour, Node, Metastasis) with a quantitative measurement of immune cell infiltration. Subsequent studies have adapted and expanded this concept to other solid tumours, incorporating additional immune populations, spatial refinement, and computational methodologies. The IS represents a composite, spatially resolved scoring system, and its interpretation is inherently context- and tumour-dependent. Additionally, it is necessary to address that originally, in colorectal cancer, the densities of CD3^+^ and CD8^+^ T cells, both in the TC and in the IM, were analysed to confirm the IS value; however, this score has been modified depending on the tissue analysed. This way, novel markers have been included, leading to a modified IS rather than a true IS as conceived for colorectal cancer. Therefore, it is important to distinguish between different categories of immune-based biomarkers that are often collectively referred to under the umbrella of “Immunoscore-like” approaches. In this review, we stratify the literature into four main groups: i) the classical IS, defined by standardised quantification of CD3^+^ and CD8^+^ T cells in the TC and IM, as originally validated in colorectal cancer; ii) adapted IS models, which preserve this spatial CD3/CD8 framework but are applied to other tumour types; iii) modified tissue-based immune scores, which expand the original model by incorporating additional immune markers (e.g., CD45RO, FOXP3, CD66b, and PD-1/PD-L1); and iv) broader immune contexture-based classifiers and surrogate biomarkers, including transcriptomic signatures, radiomic models, and systemic immune indices. These categories are biologically and methodologically distinct and should not be considered interchangeable, although they share a common conceptual basis in the characterisation of the tumour immune microenvironment.

**Figure 1:**
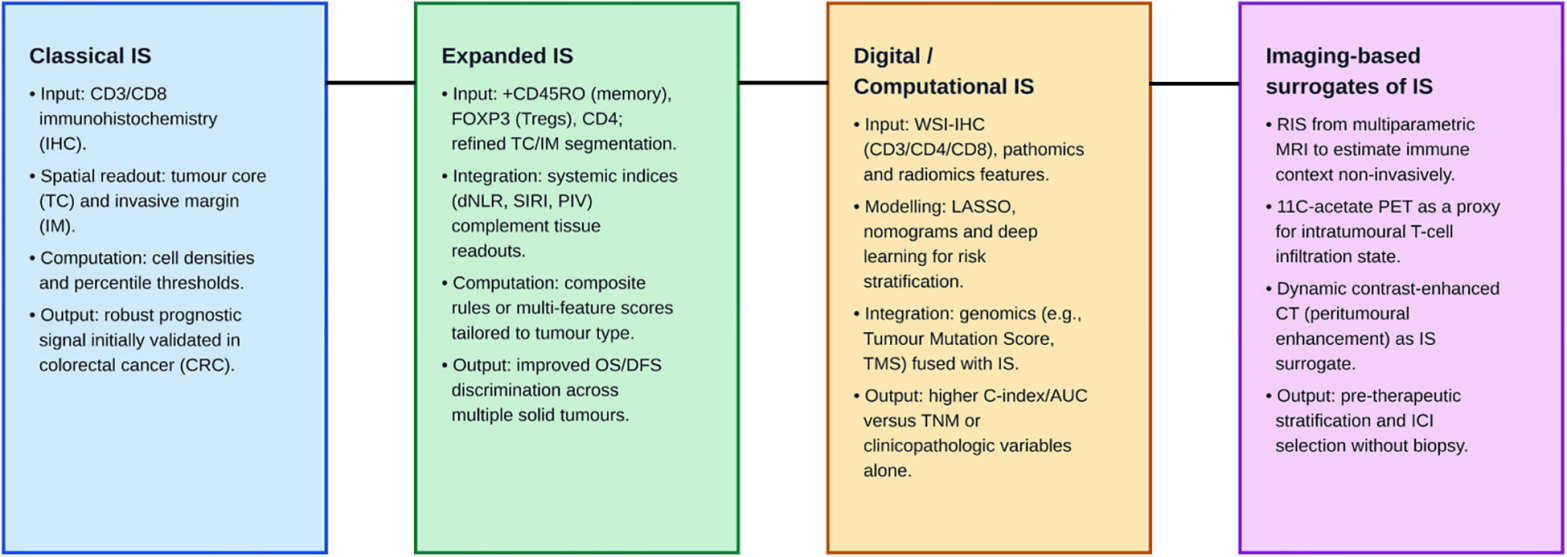
Conceptual landscape of Immunoscore (IS) and immune contexture-based biomarkers across solid tumours. Classical IS quantifies CD3^+^/CD8^+^ T cells in tumour core (TC) and invasive margin (IM) using immunohistochemistry (IHC) and percentile-based density thresholds, originally validated in colorectal cancer (CRC). Expanded IS adds CD45RO^+^, FOXP3^+^, and CD4^+^ with refined compartmentalisation and integrates systemic indices [derived neutrophil-to-lymphocyte ratio (dNLR), Systemic Inflammatory Response Index (SIRI), and Pan-Immune Inflammation Value (PIV)] to improve overall survival/disease-free survival (OS/DFS) discrimination. Digital/computational IS leverages whole-slide imaging (WSI)-IHC, pathomics/radiomics, and machine learning (ML) (e.g., LASSO, nomograms, and deep learning) and fuses genomic features [e.g., Tumour Mutation Score (TMS)], outperforming TNM/clinical models. Imaging-based surrogates estimate IS non-invasively via MRI radiomics (RIS), ^11^C-acetate PET (proxy of intratumoural T-cell engagement), and dynamic CT peritumoural enhancement, enabling pre-therapeutic stratification and Immune Checkpoint Inhibitor (ICI) selection without biopsy. These approaches represent parallel and partially overlapping strategies with varying levels of validation and standardisation. Later approaches represent conceptual extensions and are not directly equivalent to the classical IS.

Parallel advances in digital pathology, radiomics, transcriptomics, and integrative modelling have further propelled the IS from a histopathological descriptor to a multidimensional biomarker capable of informing prognosis, recurrence risk, and response to systemic therapies, including immunotherapy. This review thoroughly examines the current evidence supporting the IS across multiple solid malignancies, beyond colorectal cancer, highlighting biological insights, methodological innovations, and emerging clinical applications, whilst addressing remaining challenges to its routine implementation (**Figure 1**).

## 2 Materials and methods

This work was conducted as a narrative, non-systematic review aimed at providing a comprehensive and conceptually integrated overview of Immunoscore and related immune contexture-based approaches across solid tumours. Accordingly, the methodology was designed to ensure broad coverage of relevant literature rather than to perform a formal systematic review or meta-analysis.

A structured literature search was performed primarily in PubMed/MEDLINE, complemented by Scopus and Web of Science to enhance coverage. Google Scholar was used selectively to identify additional relevant or recently published studies not yet indexed in traditional databases. The search strategy combined controlled vocabulary (MeSH terms) and free-text keywords, including but not limited to the following: “Immunoscore”, “tumour-infiltrating lymphocytes”, “immune contexture”, “CD3”, “CD8”, “immune infiltration”, “treatment-naïve”, “early-stage cohorts” and “solid tumours”, combined with specific cancer types (e.g., “non-small cell lung cancer”, “hepatocellular carcinoma”, “gastric cancer”, “melanoma”, “head and neck cancer”, and “bladder cancer”). The isolated use of the term “IS” was avoided due to its lack of specificity.

The search focused on articles published in English within the last 10 years to capture contemporary developments in the field, although seminal earlier studies were included when conceptually relevant (e.g., foundational work on the IS and immune contexture). Eligible study types included clinical trials, prospective and retrospective cohort studies, translational research, biomarker-development studies, and high-quality review articles. Given the evolving and multidisciplinary nature of the topic, studies employing emerging methodologies—such as digital pathology, radiomics, transcriptomics, and integrative modelling—were also considered.

Study selection was performed by two independent reviewers based on relevance to the scope of the review, with emphasis on studies providing clinically or biologically meaningful insights into immune contexture and its prognostic or predictive value. Discrepancies were resolved by consensus. Reference lists of selected articles and relevant reviews were manually screened to identify additional key publications.

### 2.1 IS and non-small-cell lung cancer

Over the past decade, the story of the IS in non-small-cell lung cancer (NSCLC) has unfolded from a simple premise: clinical outcomes hinge not only on tumour size and spread but also on the immune architecture that permeates and surrounds the malignancy. Early syntheses established the immune contexture—the type, density, spatial disposition, and functional orientation of infiltrating immune cells—as a major determinant of progression and survival in NSCLC. Consistently, abundant CD8^+^ cytotoxic and CD3^+^ T-cell infiltrates—especially within stromal bands and at invasive fronts—were linked to improved prognosis, whereas FOXP3^+^ regulatory T cells and immunosuppressive myeloid compartments tracked with adverse outcomes (**Table 1**). Framed by the immunoediting paradigm (elimination, equilibrium, and escape), these observations invited the lung field to adapt the colorectal IS—a standardised quantification of intratumoural immune response—to complement and refine TNM staging in a biologically meaningful way (7) (**Table 2**).

**Table 1:** Immune-based biomarkers across cancers: conceptual classification and summary.

Tumour type	Biomarker category	Purpose	Samples and methods	Main findings	References
Non-small-cell lung cancer (NSCLC)	Adapted IS; modified tissue-based IS; composite immune biomarker	To adapt IS beyond CRC and integrate it with additional immune, systemic, and genomic features for prognostic stratification	Retrospective cohorts; IHC quantification of CD3^+^/CD8^+^ (± CD4^+^, CD45RO^+^, and CD68^+^) in TC and IM; digital pathology (IMPRS); integration with dNLR and genomic tumour mutation score	High CD3^+^/CD8^+^ densities (adapted IS) associated with improved OS/DFS; peritumoural immune exclusion linked to worse survival; composite models outperform TNM	(5–11)
Hepatocellular carcinoma (HCC)	Adapted IS; composite immune biomarker; non-invasive surrogate biomarker	To evaluate immune contexture and develop non-invasive estimators of immune infiltration and therapeutic response	Multi-omics analyses; CODEX/multiplex IHC-derived IS; MRI radiomics (RIS); PET/CT (^11^C-acetate *vs* FDG); contrast-enhanced CT (APE)	High adapted IS associated with improved survival; immunogenomic heterogeneity linked to immune escape; radiomic and imaging surrogates predicted prognosis and immunotherapy response	(12–15)
Head and neck cancer (HNSCC/OSCC/LSCC)	Adapted IS; modified tissue-based IS; composite immune biomarker	To characterise immune contexture and integrate tissue and systemic immune parameters	IHC and digital pathology; IS (CD3^+^/CD8^+^); expanded models including CD45RO^+^, FOXP3^+^; integration with SIRI/PIV	High IS associated with improved OS/DFS; immune composition and checkpoint expression refine prognosis; combined systemic and tissue markers enhance predictive accuracy	(16–21)
Gastric cancer (GC)	Modified tissue-based IS; composite immune biomarker	To develop integrative immune scores incorporating checkpoint markers and transcriptomic signatures	IHC-based composite scores (PD-1/PD-L1/CD8); LASSO-derived IS-GC (CD3^+^, CD8^+^, CD45RO^+^, and CD66b^+^); transcriptomic immune-cell signatures; biopsy-based IS	Composite IS models outperform TNM; predictive value for chemotherapy benefit; transcriptomic signatures linked to prognosis and treatment response	(22–26)
Melanoma	Composite immune biomarker; transcriptomic immune classifier	To predict immunotherapy response, TIL expansion, and survival based on immune contexture	Multispectral IHC; RNA deconvolution (CIBERSORT); gene-expression signatures; analysis of TIL subsets in primary tumours and brain metastases	Immune cell ratios (e.g., CD8:FOXP3) predict ACT success; transcriptomic IS predicts anti-PD-1 response; high TIL density associated with improved survival	(27–31)
Bladder cancer (BC)	Adapted IS; modified tissue-based IS	To evaluate the prognostic value of immune infiltration and develop IS-based models in NMIBC and MIBC	IHC quantification of CD3^+^/CD8^+^; expanded models including CD45RO^+^ and FOXP3^+^; digital pathology-derived modified IS	High stromal CD3^+^ associated with better OS; modified IS predicts progression-free and cancer-specific survival; context-dependent role of FOXP3^+^ cells	(32–34)

^18^ F-FDG PET, ^18^F-fluorodeoxyglucose positron emission tomography; ACT, adoptive T-cell therapy; APE, arterial-phase enhancement; HNSCC, head and neck squamous cell carcinoma; IHC, immunohistochemistry; IM, invasive margin; IMPRS, Immune Prognostic Risk Score; IS, Immunoscore; LSCC, laryngeal squamous cell carcinoma; MRI, magnetic resonance imaging; OSCC, oral squamous cell carcinoma; PIV, Pan-Immune Inflammation Value; SIRI, Systemic Inflammatory Response Index; TC, tumour core; TIL, tumour-infiltrating lymphocyte; TMS, tumour mutation score; dNLR, derived neutrophil-to-lymphocyte ratio; NMIBC, non-muscle-invasive bladder cancer; CRC, colorectal cancer; OS/DFS, overall survival/disease-free survival; RIS, Radiomic Immunoscore.

**Table 2:** Classification of immune contexture-based biomarkers across solid tumours according to methodological framework and level of evidence.

Reference	Tumour type	Evidence level	Marker set	Scoring framework	Validation status	IS classification
(8)	NSCLC	IV	CD3, CD8 (TC + IM)	Adapted IS (binary classification)	Internal only	IS-based
(10)	NSCLC	IV	CD8, CD45RO (TC + IM)	Modified tissue-based IS	Internal validation	IS-inspired
(11)	NSCLC	II	CD3, CD4, CD8 (IHC features)	Computational (IMPRS, ML-based)	External validation	IS-inspired
(12)	NSCLC	IV	CD3, CD8 + genomic features	Integrated genomic–immune model	Internal validation	IS-inspired
(14)	HCC	IV	CD8 + multi-omics immune features	Integrated immunogenomic model	Internal validation	IS-inspired
(15)	HCC	II	Multiplex immune markers	Radiomic Immunoscore (RIS)	External validation	IS-inspired
(16)	HCC	IV	^11^C-acetate + ^18^F-FDG	PET/CT	Internal validation	IS-inspired
(17)	HCC	IV	CD3, CD8 + CECT	CECT	Internal validation	IS-inspired
(18)	HNSCC	IV	CD3, CD8, PD-1/PD-L1	Adapted IS + checkpoint profiling	Internal validation	IS-inspired
(19)	HNSCC	IV	CD3, CD8 (TC + IM)	Adapted IS (0–4 scale)	Internal validation	IS-based
(20)	OSCC	III	CD3, CD8, CD45RO, FOXP3	Composite immune score (7 features)	External cohorts	IS-inspired
(21)	LSCC	IV	CD3. CD4, CD8 + TIL density	SIRI score	Internal cohort	IS-inspired
(22)	ESCC	IV	CD3, CD8 + systemic markers	Integrated IS + PIV model	Internal validation	IS-inspired
(23)	Salivary gland cancer	IV	CD3, CD8, FOXP3, CD68, CD163	Immune infiltration analysis	Internal validation	IS-inspired
(24)	Gastric cancer	III	CD3, CD8, PD-1, PD-L1	IHC	2 independent cohorts	IS-inspired
(25)	Gastric cancer	IV	CD3, CD8, PD-L1 + microsatellite instability	Immune infiltration analysis	Internal validation	IS-inspired
(26)	Gastric cancer	III	CD3, CD8, CD45RO, CD66b	Composite Immunoscore (IS-GC)	External and internal validation	IS-inspired
(27)	Gastric cancer	IV	CD3, CD8 + immune cell fractions	Lasso Cox regression model	2 independent cohorts	IS-inspired
(28)	Gastric cancer	IV	CD3, CD8, CD45RO, FOXP3, Granzyme B	IHC digitally quantified	Internal validation	IS-inspired
(29)	Melanoma	IV	CD3, CD8, FOXP3, CD163, PD-L1	Multispectral fluorescent IHC	Internal validation	IS-inspired
(30)	Melanoma	IV	Transcriptomic immune subsets	RNA-based Immunoscore	External validation	IS-inspired
(33)	Melanoma (brain metastases)	IV	CD3, CD8, CD45RO, FOXP3	Composite TIL-based score	Internal validation	IS-inspired
(34)	Bladder cancer	IV	CD3, CD8 (TC and IM)	Adapted IS (binary classification)	Internal validation	IS-based
(35)	Bladder cancer	IV	CD3, CD8, CD45RO	IHC	Internal validation	IS-inspired
(36)	Bladder cancer	IV	CD3/CD8/FOXP3, CD45RO	IHC	Internal validation	IS-inspired

TC, tumour core; IM, invasive margin; IS, Immunoscore; ML, machine learning; PIV, Pan-Immune Inflammation Value; CECT, contrast-enhanced computed tomography; LSCC, laryngeal squamous cell carcinoma; NSCLC, non-small-cell lung cancer; IHC, immunohistochemistry; IMPRS, Immune Prognostic Risk Score; HNSCC, head and neck squamous cell carcinoma; OSCC, oral squamous cell carcinoma; TIL, tumour-infiltrating lymphocyte; SIRI, Systemic Inflammatory Response Index; ESCC, oesophageal squamous cell carcinoma.

The natural next question was whether prominent immune biomarkers, notably PD-L1, may substitute for a granular reading of the microenvironment. In 2017, Ishii and colleagues examined resected stage II–III NSCLC patients receiving adjuvant chemotherapy, quantifying PD-L1 and a binary IS based on CD3/CD8 densities in the TC and IM. PD-L1 positivity (~61%) associated with higher IS, yet PD-L1 alone did not predict disease-free or overall survival; by contrast, IS 3–4 and high stromal CD8^+^ density coincided with fewer recurrences and longer disease-free survival (DFS). In other words, the geography of intratumoural T cells (simultaneously present and organised in the TC and IM) carried more prognostic weight than epithelial PD-L1 expression per se (8).

Building on that insight, a more recent study broadened both what and where to measure. Modified tissue-based immune scores expanded the original IS framework beyond CD3/CD8 to incorporate CD4^+^ T cells and CD68^+^ macrophages and crucially distinguished intratumoural from peritumoural compartments in chemo-naïve stage II–III NSCLC (9). This spatial refinement proved revealing: high PT immune infiltration, effector cells marshalling outside tumour nests without penetration, was associated with worse 3-year survival, consistent with an immune-exclusion phenotype. Moreover, adding a systemic metric, the derived neutrophil-to-lymphocyte ratio (dNLR), further improved discriminatory power, underscoring how tissue–blood composites can narrate risk more completely than either dimension alone (9).

In 2021, the modified tissue-based IS matured into a clinically persuasive instrument in a completely resected stage IIIA (N2) NSCLC. CD45RO^+^ memory and CD8^+^ cytotoxic T cells were quantified across the TC and IM, closely mirroring original colorectal IS methodology (10). Patients with IS 3–4 experienced significantly longer distant metastasis-free and overall survival than those with IS 0–2 [5-year overall survival (OS), 45% *vs* 28%; *p* = 0.001], with emerging CD45RO^+^ density in the IM as the single strongest predictor (10). Biologically, this points to the effector memory patrol at the tumour edge as the locus of durable surveillance against micrometastatic escape following surgery.

From 2022 onward, the narrative becomes decisively computational and multimodal, without losing biological footing. A digital image-based Immune Prognostic Risk Score (IMPRS) was devised, extracting 5,580 quantitative features from immunohistochemistry (IHC) whole-slide images of CD3/CD4/CD8 in the TC/IM, selecting an eight-feature signature via Least Absolute Shrinkage and Selection Operator (LASSO) Cox, and integrating it with age, T-stage, and N-stage into a nomogram (11). Across discovery and external validation cohorts (n = 283), IMPRS outperformed clinicopathological models for overall survival (C-index up to 0.869), with robust calibration and superior net benefit; importantly, prognostic signal persisted in stage I disease and irrespective of adjuvant chemotherapy (11). In essence, computational immunomorphometry captured actionable signals that outstrip conventional vision and statistics.

In parallel, a genomic thread into the fabric of adapted IS was stitched by integrating an 11-gene Tumour Mutation Score (TMS) (e.g., MUC4, ZFHX4, and MEOX2) with a four-tier IS (CD3^+^/CD8^+^ in the TC/IM) to predict early recurrence in stage I NSCLC (12). Strikingly, high TMS and low IS were independent risk factors, whereas PD-L1 and epithelial growth factor receptor (EGFR) status carried no prognostic weight. Importantly, the combined nomogram achieved an area under the curve (AUC) of 0.935/0.932 (training/testing) with excellent calibration. This highlights the complementarity of genomic burden and immune infiltration precisely where adjuvant decisions are most fraught, early-stage disease (12).

Although the focus is on lung primary tumours, the importance of modified IS was also observed in colorectal cancer lung metastases. In this context, pathomics (deep learning on histopathology), radiomics (quantitative imaging), and the IS were integrated, showing that pathomics/radiomics scores correlated inversely with the IS, each component conferred independent prognostic value, and the combined nomogram outperformed single-modality models (3-year AUC 0.860 for OS; 0.875 for DFS), with clinical utility (13). Beyond site specificity, the message is that hybrid immune-morphometric models that fuse spatial immunometry with image analytics refine postoperative risk and enable more tailored surveillance and therapy.

Taken together, the IS (and their modifications) in NSCLC showed that where T cells are (TC and, especially, IM), what lineage they represent (cytotoxic *vs* memory), and whether they are blocked at the periphery (immune exclusion) together anticipate the clinical course. Moreover, algorithmic integration of digital immune features and genomic risk markedly enhances predictive capacity. To translate this into routine practice, the next steps are standardisation (markers, compartments, and thresholds), prospective consensus IS validation, and harmonised digital workflows linked to concrete adjuvant and immunotherapy decisions (7–12).

### 2.2 IS and hepatocellular carcinoma

Traditional anatomical staging (AJCC/UICC TNM) cannot fully explain why patients with ostensibly similar tumour burdens experience vastly different outcomes after resection. Hepatocellular carcinoma (HCC) (often arising in chronically inflamed livers) exposes the limits of morphology alone and elevates the importance of immune contexture: the type, density, and spatial distribution of TILs. Building on the colorectal cancer precedent, the IS quantifies CD3^+^ and CD8^+^ T cells within the TC and IM to generate a four-point scale (0–4) that can outperform pure anatomy in predicting recurrence and survival (**Table 1**). The central question for liver oncology has become how to adapt and operationalise the IS for HCC, and critically, how to estimate the IS non-invasively so clinicians can select and sequence immunotherapy without mandating tissue sampling (**Table 2**).

In a study performing a multi-lesion, multi-omics atlas across 47 tumours, intrahepatic metastasis Intrahepatic metastasis from multicentric occurrence (MO) was distinguished (14). Lesions exhibited sparse CD8^+^ infiltration, M2 macrophage skewing, lower adapted IS, and antigen presentation defects such as HLA loss of heterozygosity (LOH). MO lesions showed denser CD8^+^ fronts, higher IS, and adaptive resistance via PD-1/PD-L1 engagement. Integrating the IS, T-cell receptor (TCR) clonality, immunoediting, and HLA-LOH stratified patients into low- *vs* high-immune-evasion phenotypes with distinct recurrence risks and refined prognosis by capturing how effectively tumours are recognised and attacked by host immunity (14).

More recently, a radiomic model of TME status within the tumour was evaluated to predict prognosis and immunotherapy response in 301 patients who underwent magnetic resonance imaging (MRI) examinations (15). CODEX multiplex immunophenotyping (17 markers, including CD3, CD8, PD-1/PD-L1, CD68, and HLA-DR) was analysed on Formalin-Fixed, Paraffin-Embedded (FFPE) samples to construct a histology-anchored IS via LASSO Cox (15). A Radiomic Immunoscore (RIS) from 6,115 multiparametric MRI descriptors was also developed. The RIS model showed the capability of predicting therapeutic response to anti-programmed cell death 1 (anti-PD-1) immunotherapy in an independent cohort of advanced HCC patients (area under the curve = 0.731) (15). This way, the integrated RIS model exhibited not only higher accuracy in predicting prognosis but also the potential guiding significance to HCC immunotherapy.

Later, ^11^C-acetate *vs* fludeoxyglucose F-18 (^18^F-FDG) PET/CT was prospectively compared in 32 resectable HCC cases (14). ^11^C-Acetate was more sensitive than ^18^F-FDG for detecting immune infiltration (88% *vs* 58%), and its uptake correlated with higher CD3^+^/CD8^+^ densities in the TC and IM and, subsequently, with higher adapted IS. Patients with acetate-avid tumours (also higher adapted IS) experienced longer overall survival (*p* = 0.002), with the only-acetate-avid subset faring best, whereas only-FDG-avid fared worst. Notably, adapted IS rose with acetate uptake but not with FDG, implying that acetate metabolism tracks functional, cytotoxic T-cell engagement and can serve as a non-invasive gauge of the “effector IS state” (16). This way, it could be hypothesised that ^11^C-acetate PET/CT can help pre-select “immune-inflamed” HCCs for checkpoint monotherapy or guide combination escalation where effector signals are present.

In a very recent study, it was tested whether peritumoural arterial-phase enhancement (APE) on contrast-enhanced computed tomography (CECT) predicted IS (17). In 96 resected HCCs, APE independently associated with the tissue-adapted IS (CD3^+^/CD8^+^ in the TC/IM, 0–4 scale; *p* = 0.004). In a validation cohort (81 nodules treated with atezolizumab + bevacizumab or durvalumab + tremelimumab), the presence of peritumoural enhancement heralded significantly longer time to nodular progression (median undefined *vs* 180 days; *p* < 0.001), signalling better immunotherapy responsiveness when CECT-inferred IS was high. Mechanistically, the feature likely reflected compensatory arterial hyperperfusion (portal microthrombi) and correlated with an “immune-excluded” pattern on gadoxetate disodium-enhanced MRI (EOB-MRI), yet clinically marked an immune-rich milieu amenable to anti-PD-L1 + anti-VEGF combinations. This way, APE could be a practical, widely accessible imaging cue for high IS/immune-inflamed biology, useful when MRI or PET is unavailable (17).

Across imaging modalities, adapted IS (CD3^+^/CD8^+^ in the TC/IM) persists as a prognostic and predictive axis, yet could now be estimated without tissue. These tools collectively decrease dependence on biopsy, mitigate sampling error and bleeding risk, and enable pre-therapeutic immune profiling that is scalable across hospital settings. This way, the IS in HCC has moved from histology to imaging, enabling clinicians to see the immune landscape before treatment. MRI radiomics (RIS), ^11^C-acetate PET, and arterial-phase CECT are complementary windows into the same biology, where the T cells are localised (TC *vs* IM), how effective they are (granzyme B and effector signals), and whether antigen presentation is intact (HLA-LOH). Used together, they convert modified IS from a static count into a clinical map, guiding the right therapy to the right immune phenotype and advancing precision immuno-oncology in liver cancer.

### 2.3 IS and head and neck squamous cell carcinoma

Head and neck squamous cell carcinoma (HNSCC), including oral squamous cell carcinoma (OSCC), remains clinically challenging due to heterogeneous outcomes within identical TNM stages. Increasing evidence highlights the prognostic relevance of TILs, particularly T lymphocytes, and their spatial distribution (**Table 1**). Building on the IS concept pioneered in colorectal cancer, recent studies have explored immune-based classifiers in HNSCC and OSCC, integrating quantitative pathology and immune checkpoint profiling to refine prognostication and inform therapeutic strategies (**Table 2**).

Although oesophageal squamous cell carcinoma (ESCC) and major salivary gland malignancies are not strictly classified within HNSCC, their inclusion in this section is conceptually justified from an immune-oncology perspective. Firstly, ESCC shares key biological and immunological features with HNSCC, including squamous histology, common carcinogenic exposures (e.g., tobacco and alcohol), and a highly inflamed tumour microenvironment characterised by variable T-cell infiltration and immune checkpoint activation. Indeed, recent studies have demonstrated that IS-based or modified immune-contexture models retain strong prognostic and predictive value in ESCC, particularly when integrated with systemic inflammatory indices, supporting the translatability of these approaches across anatomically distinct but biologically related squamous malignancies (22).

Secondly, the inclusion of major salivary gland cancers reflects the broader aim of this review to evaluate the applicability of immune contexture-based biomarkers beyond canonical indications. While these tumours are histologically and molecularly heterogeneous, emerging evidence indicates that TILs and tumour-associated macrophages (TAMs) play a critical role in disease progression, particularly in predicting lymph node metastasis and clinical outcome (23). This reinforces the notion that immune infiltration patterns represent a conserved biological dimension across diverse epithelial malignancies, even when traditional anatomical classifications differ.

Importantly, this approach is consistent with the conceptual framework of the IS as a biology-driven rather than anatomy-restricted biomarker, whose relevance lies in capturing the functional state of the tumour–immune interaction. By including ESCC and salivary gland cancers, we aimed to illustrate the generalizability and current limitations of immune contexture-based models across related tumour entities, rather than to imply equivalence with classical HNSCC. Nevertheless, we acknowledged the heterogeneity of these diseases and have interpreted the findings within their specific biological contexts.

Lechner et al. (2017) provided one of the earliest comprehensive characterisations of T-cell subsets and immune checkpoint molecules in treatment-naïve HNSCC patients (18). Their analysis revealed a predominance of effector memory T cells within tumours and a marked reduction in naïve T cells compared to peripheral blood. Regulatory T cells (Tregs) were significantly enriched in the tumour microenvironment, contributing to an immunosuppressive milieu. Importantly, PD-1 and PD-L1 expression was markedly elevated on intratumoural T cells, alongside increased CTLA-4 expression, consistent with T-cell exhaustion. Human Papillomavirus (HPV)-positive tumours exhibited higher overall lymphocyte infiltration but similar subset composition compared to HPV-negative tumours. Digital IHC enabled automated, adapted IS classification, linking high IS to increased MHC class I expression and potential responsiveness to checkpoint blockade (18).

More recently, the IS was validated in 88 surgically treated HNSCC patients, demonstrating its strong prognostic power (19). High IS (3–4) was associated with significantly improved OS and DFS compared to low scores (0–2), outperforming TNM staging in multivariate analysis. Notably, the IS refined risk stratification within identical TNM stages: advanced-stage patients with high IS achieved survival comparable to that of early-stage counterparts, whereas low-score patients fared poorly. Treatment-response analysis suggested predictive utility, as high IS correlated with better outcomes following multimodal therapy (19).

In another study, advanced prognostic modelling by developing a seven-feature immune-based score (7IFBPS) in OSCC was carried out (20). Incorporating CD3, CD8, CD45RO, and FOXP3 densities across tumour core and invasive margin, this composite score consistently predicted superior OS and DFS across multiple cohorts. Interestingly, FOXP3^+^ TILs (traditionally considered immunosuppressive) correlated positively with survival, suggesting context-dependent roles and cooperative interactions with effector subsets (20). This integrative approach exemplifies next-generation immune classifiers, offering enhanced granularity for patient stratification.

Recent studies have extended immune-based prognostic tools beyond OSCC. Systemic and local immunity in laryngeal squamous cell carcinoma (LSCC) were integrated by combining adapted IS with the Systemic Inflammatory Response Index (SIRI) (21). High adapted IS predicted favourable outcomes, whilst high SIRI correlated with poor prognosis. Patients with low SIRI and high adapted IS achieved the best survival, underscoring the interplay between systemic inflammation and local immune infiltration (21).

In oesophageal squamous cell carcinoma, a study introduced a dual-parameter model combining a modification of the IS with the Pan-Immune Inflammation Value (PIV), a blood-based marker of systemic inflammation (22). This integrated approach achieved an AUC of 0.82 for predicting 36-month OS. Patients with high modified IS and low PIV exhibited optimal pathological response and survival following neoadjuvant chemoimmunotherapy (22).

Interestingly, TIL and TAM densities were explored in major salivary gland cancers, revealing strong associations between immune infiltration patterns and lymph node metastasis (23). High densities of CD3^+^, CD8^+^, and FOXP3^+^ TILs, along with CD68^+^ and CD163^+^ macrophages, predicted nodal spread, suggesting that immune profiling could inform surgical decision-making (23).

Across these studies, a unifying theme emerges: immune contexture, quantified through the IS or composite immune-feature models, could be a potential complement to TNM staging alone. Incorporating additional markers (e.g., FOXP3 and CD45RO) and systemic indices (SIRI and PIV) further enhances predictive power. Future priorities include multicentre validation, standardisation of scoring algorithms, and integration with genomic and functional immune metrics to enable precision oncology in head and neck cancers.

### 2.4 IS and gastric cancer

Gastric cancer (GC) remains one of the most lethal malignancies worldwide, ranking amongst the top causes of cancer-related mortality. Despite advances in surgical and systemic therapy, patient outcomes remain highly heterogeneous, even within identical TNM stages. This variability reflects the biological complexity of GC, which traditional staging systems fail to capture. In response, research has shifted towards immune-based classifiers that integrate the tumour immune microenvironment into prognostic and predictive models (**Table 1**). Building on the IS concept validated in colorectal cancer, recent studies have explored its application in GC, incorporating immune checkpoint profiling and advanced computational modelling to refine risk stratification and guide therapy (**Table 2**).

Initial efforts to link immune infiltration with GC prognosis focused on individual markers such as CD8^+^ T cells or PD-L1 expression. However, these approaches yielded inconsistent results due to the multifaceted nature of antitumour immunity. This limitation was firstly addressed by developing a four-factor modified IS system integrating PD-L1 expression in tumour and immune cells, PD-1 expression in immune cells, and CD8^+^ T-cell density (24). In two independent cohorts of stage II–III GC patients, this composite score stratified patients into distinct risk groups with significantly different OS, achieving an AUC of 0.856 for 5-year mortality prediction. Patients with PD-L1^+^ tumour cells and abundant CD8^+^ infiltration exhibited the best prognosis (24), suggesting that PD-L1 expression in an inflamed microenvironment reflects adaptive immune resistance rather than immune escape.

Microsatellite instability-high (MSI-H) GC represents a molecular subtype characterised by dense lymphocytic infiltration and heightened immunogenicity. A study demonstrated that PD-L1 expression alone lacked prognostic significance in MSI-H tumours (23); however, its combination with adapted IS revealed four biologically distinct subgroups. Patients with PD-L1^+^ tumours and high adapted IS had the most favourable outcomes, whereas PD-L1^+^/IS-low tumours exhibited poor survival (25). These findings underscore the importance of contextualising checkpoint expression within immune density, with implications for predicting response to PD-1/PD-L1 blockade.

Important advances in the field were obtained by applying LASSO Cox regression to identify the most informative immune features amongst 27 candidates, constructing a five-feature composite IS for GC (IS-GC): CD3^+^ (TC and IM), CD8^+^ (IM), CD45RO^+^ (TC), and CD66b^+^ (IM) (26). In a multi-cohort study of 879 patients, high composite IS-GC predicted superior 5-year OS (48.8% *vs* 6.7%; *p* < 0.001) and DFS, independent of TNM stage. Importantly, composite IS-GC also functioned as a predictive biomarker: stage II–III patients with high composite IS-GC derived significant benefit from adjuvant chemotherapy, whereas low-score patients did not. Nomograms integrating composite IS-GC and clinicopathological factors demonstrated excellent calibration and clinical utility (26).

More recently, an eight-cell immune signature derived from transcriptomic profiling was introduced, incorporating activated CD4^+^ and CD8^+^ T cells, dendritic cells, NK cells, Th17 cells, and B-cell subsets (27). Low composite IS correlated with favourable prognosis and enhanced chemosensitivity, whilst high composite IS predicted selective benefit from Xeloda plus cisplatin over 5-fluorouracil (FU)-based regimens. Correlative analyses linked low composite IS to higher expression of immune checkpoints and interferon-γ, suggesting potential responsiveness to immunotherapy (27).

Complementing these findings, the feasibility of a biopsy-based composite IS in 131 paired biopsies and resections was evaluated (28). Despite modest concordance due to spatial heterogeneity, a composite score combining CD8^+^ and FOXP3^+^ retained prognostic significance (Hazard Ratio (HR) = 3.40; *p* = 0.015) (28), paving the way for pre-treatment immune profiling and personalised therapy.

Across diverse methodologies, from IHC to transcriptomics and digital pathology, immune contexture consistently complemented TNM staging in prognostic accuracy. Composite IS not only refines risk stratification but also predicts therapeutic benefit, bridging the gap between molecular insight and clinical action. Future priorities include prospective validation, algorithm harmonisation, and integration of the IS into immunotherapy decision-making, particularly for MSI-H and Epstein-Barr virus (EBV)-positive GC.

### 2.5 IS and melanoma

Although a standardised, consensus IS analogous to that established in colorectal cancer has not yet been formally validated in melanoma, this tumour type represents one of the most informative models for studying immune contexture-based biomarkers. Melanoma is characterised by high immunogenicity, abundant TILs, and marked sensitivity to immune checkpoint blockade, making it particularly suitable for evaluating the principles underlying the IS. In this context, most available studies correspond not to the classical IS framework but to modified tissue-based immune scores or broader immune-contexture classifiers, which integrate spatial, phenotypic, and functional dimensions of the immune infiltrate. Therefore, the melanoma literature should be interpreted as an extension of the IS concept rather than a direct application of the canonical CD3/CD8-based scoring system (26, 30, 31). To improve conceptual clarity, melanoma studies can be stratified into three main categories: i) tissue-based immune metrics reflecting IS principles (e.g., CD8^+^/FOXP3^+^ ratios and spatial TIL distribution), ii) transcriptomic or computational immune scores representing systemic or global immune contexture, and iii) functional immune readouts, such as TIL expansion capacity or response to immune checkpoint inhibitors. While these approaches differ methodologically, they converge on a common biological axis: the balance between effector and suppressive immune components and their spatial organisation within the tumour microenvironment.

Cutaneous melanoma is amongst the most aggressive cancers, characterised by high metastatic potential and poor survival despite advances in immunotherapy. Central to disease progression and therapeutic response is the TME, particularly the composition and functional state of TILs (**Table 1**). Recent evidence demonstrates that the balance and spatial distribution of immune subsets—not their absolute abundance—dictates clinical outcomes (**Table 2**).

Adoptive T-cell therapy (ACT) depends on generating autologous tumour-reactive TILs; yet up to 45% of melanoma samples fail to yield such cultures under standard protocols. This challenge was firstly addressed by integrating multispectral IHC with comparative culture strategies (29). Using seven-colour panels (CD3^+^, CD8^+^, FoxP3^+^, CD163^+^, PD-L1^+^, and melanoma markers), it was demonstrated that combining enzymatic digestion with tumour fragment culture increased TILs recovery to 92%, compared to ~77% with either method alone. Crucially, immune context (not absolute cell counts) predicted success: a high CD8:FoxP3 ratio correlated strongly with TIL expansion [positive predictive value (PPV) 91%, negative predictive value (NPV) 86%], and incorporating CD8:PD-L1 improved negative predictive value to 100% (29). These results underscore that regulatory T cells and checkpoint ligand expression modulate *ex vivo* T-cell proliferation, suggesting that pre-culture immune profiling could optimise ACT workflows. These findings also align with IS principles, as the relative abundance and balance of cytotoxic versus regulatory T-cell populations (e.g., CD8:FOXP3 ratio) represent a functional extension of immune contexture metrics beyond simple density measurements (29).

To anticipate response to anti-PD-1 therapy, an RNA-based IS using CIBERSORT deconvolution of 22 immune subsets across six melanoma cohorts was developed (26). A LASSO logistic model incorporating eight cell types (e.g., naïve/memory B cells, follicular helper T cells, Tregs, and eosinophils; negative weights for M0 macrophages, plasma cells, and γδ T cells) achieved AUCs of 0.77–0.80 in validation and 0.73 in on-treatment samples (30). High-score tumours exhibited superior objective response rates (ORR 53.8% *vs* 17.7% pre-therapy; 66.7% *vs* 16.7% in neoadjuvant cohorts; *p* < 0.001 in both) and trends towards improved progression-free survival and OS. Mechanistically, high scores correlated with increased TIL fractions, IFN-γ signalling, and enrichment of antigen presentation and PD-L1 pathways, reflecting a pre-existing immune-active state amenable to checkpoint reinvigoration (30). Although derived from transcriptomic deconvolution rather than histopathology, this RNA-based IS represents a systemic and functional analogue of tissue IS, capturing the coordinated immune landscape that underlies response to checkpoint blockade (30).

Beyond these studies, conventional and advanced TIL profiling approaches were reviewed, highlighting the prognostic relevance of CD8^+^ cytotoxic T cells, FOXP3^+^ Tregs, and CD20^+^ B cells (31). Multiplex immunofluorescence, imaging mass cytometry, and CODEX enabled high-dimensional spatial mapping of immune contexture, promising standardised prognostic tools and improved predictive accuracy for immunotherapy (31). Complementing this, a seven-gene prognostic signature in melanoma was created (32). Melanoma patients from The Cancer Genome Atlas (TCGA) were divided into a low-risk group and a high-risk group using the median risk score. Receiver Operating Characteristic (ROC) analysis for OS showed that the AUC was 0.701 for 1 year, 0.726 for 3 years, and 0.745 for 5 years. Moreover, a nomogram was constructed for individualised risk prediction, and the AUC was 0.829 for 3 years and 0.803 for 5 years (32).

However, historically considered “immune-privileged”, the central nervous system (CNS) presents unique challenges for immunotherapy. A total of 116 brain metastases (BMs) from melanoma and other primaries were analysed, quantifying TIL subsets (CD3, CD8, CD45RO, FOXP3, and PD-1) and PD-L1 expression (33). Contrary to prior assumptions, 99% of BMs contained TIL, with melanoma lesions exhibiting the highest densities. Importantly, elevated CD3^+^, CD8^+^, and CD45RO^+^ infiltration, and a high composite IS were independently associated with prolonged survival (median OS 27 *vs* 10 months, high *vs* low score, respectively). PD-L1 was detected in ~28% of cases but did not correlate with TIL density. An intriguing observation linked CD8^+^ abundance to peritumoural oedema, suggesting radiographic inflammation as a surrogate for immune activation (33). These results position the IS as a robust prognostic tool even in CNS disease and support extending checkpoint blockade strategies to patients with BM. Notably, the association between high TIL density and improved survival in brain metastases further supports the robustness of immune contexture as a prognostic axis, consistent with IS-based observations across solid tumours.

Across methodologies (multiplex IHC, spatial histology, and transcriptomic modelling), a unifying principle emerges: immune equilibrium governs melanoma outcomes. Ratios such as CD8:FoxP3 and composite IS integrated effector and suppressive signals, predicting the feasibility of ACT, prognosis in brain metastases, and responsiveness to PD-1 blockade. Future directions include harmonising spatial proteomics with genomic models to enable multi-parametric immune profiling, guiding patient stratification and rational combination therapies in melanoma.

### 2.6 IS and bladder cancer

Bladder cancer (BC) remains a clinically heterogeneous disease with an unpredictable course, posing significant challenges for patient management. Although TNM staging constitutes the cornerstone of prognostic assessment, it does not fully account for the biological complexity of the TME, particularly its immune component. TILs have emerged as key determinants of tumour behaviour, influencing disease progression and therapeutic response (**Table 1**). In this context, IS-based models have gained increasing attention as potential tools for improving prognostic stratification and advancing precision oncology (**Table 2**).

The prognostic relevance of immune cell localisation was investigated in 167 pT1 BC patients, demonstrating that immune contexture provides clinically meaningful information beyond conventional staging (34). Using IHC for CD3^+^ and CD8^+^, the authors showed that high stromal infiltration of CD3^+^ T cells was associated with improved OS, particularly in high-grade (G3) tumours (*p* = 0.01). In contrast, a high density of CD8^+^ T cells at the IM was associated with poorer outcomes. These findings highlighted the importance of the spatial distribution and functional context of immune infiltrates. Furthermore, tertiary lymphoid structures and lymphocyte aggregates were more frequently observed in high-grade tumours, suggesting an activated yet potentially dysregulated immune response (34). Collectively, these results supported the development of an IS specifically adapted to non-muscle-invasive bladder cancer (NMIBC), with potential implications for risk stratification and follow-up strategies.

Building on this concept, immune profiling was expanded to include CD45RO^+^ memory T cells and FOXP3^+^ Tregs (35). In a cohort of 85 BC cases, tumours associated with early progression or muscle invasion exhibited increased densities of CD3^+^, CD8^+^, and CD45RO^+^ cells, reflecting an active immune response against tumour antigens. However, with advancing disease, an increased presence of FOXP3^+^ Tregs and inhibitory immune checkpoints was observed, indicating a shift towards an immunosuppressive TME. Although no significant associations with OS were identified, the study provided evidence of a dynamic balance between effector and regulatory immune mechanisms during BC progression (35).

Further refinement of immune-based prognostication was achieved by introducing a modified IS integrating CD3^+^, CD8^+^, CD45RO^+^, and FOXP3^+^ cell densities quantified by digital pathology (36). In a cohort of 159 patients undergoing radical cystectomy, a high modified IS (mIS) was significantly associated with improved progression-free survival and cancer-specific survival, particularly in patients with AJCC stage IIIa disease [HR for cancer-specific survival (CSS), 3.5; *p* = 0.006]. Notably, FOXP3 expression (traditionally considered a marker of immunosuppression) emerged as a strong predictor of favourable outcome, challenging established assumptions regarding the role of Tregs in BC (36). These findings underscore the value of integrated immune profiling as a prognostic tool that complements TNM staging.

Taken together, these studies demonstrate that immune contexture, defined by the density, phenotype, and spatial distribution of TILs, provides prognostic information that is not captured by conventional staging systems alone. Standardisation and validation of IS methodologies may facilitate their transition from research settings into routine clinical practice, enabling more precise risk stratification, treatment selection, and surveillance strategies. The immune microenvironment thus represents a central component of BC biology with significant translational potential.

### 2.7 IS and IS-based framework limitations

While the IS framework has demonstrated prognostic and predictive value across multiple solid tumours by quantifying the densities of CD3^+^ and CD8^+^ T cells in the TC and IM, it is increasingly recognised that density-based metrics alone may not fully capture the biological complexity of the tumour immune microenvironment. In particular, the distinction between immune-inflamed (infiltrated) and immune-excluded phenotypes represents a critical dimension of tumour–immune interaction with important clinical implications (37). Immune-inflamed tumours are characterised by the presence of effector T lymphocytes within the tumour parenchyma, often accompanied by interferon-γ signalling, antigen presentation, and expression of immune checkpoints such as PD-1/PD-L1. These tumours typically exhibit a “hot” microenvironment and are associated with a favourable prognosis and increased responsiveness to immunotherapy. In contrast, immune-excluded tumours display abundant immune cells that are spatially restricted to the stromal compartment or invasive margin, with limited penetration into tumour nests (37). Despite sometimes exhibiting high overall lymphocyte density, these tumours reflect a fundamentally different biological state in which physical, molecular, or stromal barriers, such as aberrant vasculature, extracellular matrix remodelling, TGF-β signalling, or myeloid-driven immunosuppression, impede effective T-cell trafficking and tumour cell contact (38).

Importantly, conventional IS captures this dimension only partially. Although the inclusion of both the TC and IM compartments introduces a degree of spatial resolution, the scoring system does not explicitly distinguish between true intratumoural infiltration and stromal confinement (39). As a result, tumours with similar IS values may represent biologically distinct entities with divergent clinical behaviour. This limitation has prompted the development of next-generation immune classifiers incorporating spatial metrics, such as multiplex imaging or digital pathology. These approaches enable more precise characterisation of immune architecture, including the identification of immune-excluded, immune-desert, and immune-inflamed phenotypes within a unified framework.

From a clinical perspective, distinguishing between immune infiltration and exclusion is particularly relevant for therapeutic decision-making. Immune-inflamed tumours are more likely to respond to immune checkpoint inhibitors, whereas immune-excluded tumours may require combination strategies aimed at overcoming stromal barriers or reprogramming the tumour microenvironment (40). Therefore, integrating spatial immune context into IS-based models could further enhance their predictive accuracy and clinical utility.

An additional conceptual limitation of density-based immune metrics such as the IS is their inherently static nature, which may not fully reflect the temporal and context-dependent dynamics of tumour–immune interactions. The TME is a highly dynamic ecosystem that evolves throughout tumour progression and under therapeutic pressure, shaped by processes such as immunoediting (elimination, equilibrium, and escape) (41). During early stages, effective cytotoxic and memory T-cell responses may constrain tumour growth, typically corresponding to an immune-inflamed phenotype associated with a favourable prognosis. However, over time, selective pressures can promote immune evasion mechanisms, including T-cell exhaustion, upregulation of immune checkpoints, loss of antigen presentation (e.g., HLA alterations), and expansion of immunosuppressive populations such as regulatory T cells and tumour-associated macrophages (41). Consequently, the functional role and prognostic significance of specific immune subsets may shift across disease stages and tumour types. For example, whilst high CD8^+^ T-cell density is generally associated with improved outcomes, its prognostic value may be attenuated or even reversed in contexts where these cells exhibit an exhausted phenotype or are spatially restricted (42). Similarly, FOXP3^+^ regulatory T cells, traditionally linked to immunosuppression, have demonstrated context-dependent associations with both favourable and adverse prognosis depending on tumour type and immune composition (43). Moreover, therapeutic interventions—including chemotherapy, radiotherapy, and immunotherapy—can profoundly remodel the TME, altering both the quantity and functional orientation of immune infiltrates over time (44). These observations highlight that immune contexture is not a fixed attribute but a dynamic process and suggest that longitudinal assessment strategies, integration of functional immune markers (e.g., activation/exhaustion signatures), and incorporation of temporal data may be required to fully capture the complexity of tumour–immune interactions. In this regard, next-generation IS-based models integrating transcriptomic, spatial, and longitudinal data may provide a more comprehensive and clinically relevant representation of antitumour immunity.

A further limitation lies in the nature of the current evidence base supporting the IS and related immune contexture-based biomarkers. Much of the literature is derived from retrospective, single-centre cohorts, often with limited sample sizes and heterogeneous patient populations in terms of stage, treatment exposure, and molecular background. This variability, together with the frequent use of cohort-specific thresholds (e.g., median or percentile-based cut-offs), hampers comparability across studies and limits generalizability. In addition, methodological heterogeneity, including differences in immune markers, staining protocols, and definitions of tumour compartments such as the tumour core and invasive margin, remains a major barrier to standardisation and clinical implementation (1, 2, 39).

Additionally, the increasing incorporation of computational approaches, including radiomics, pathomics, and transcriptomic modelling, has further expanded the scope of IS-like frameworks but also introduced additional challenges. Many of these models are developed in relatively small datasets with high-dimensional features and are frequently validated only internally, raising concerns about overfitting and inflated performance estimates. The lack of robust external validation across independent cohorts and institutions limits confidence in their reproducibility and clinical applicability (11, 15).

Moreover, the biological interpretation of immune contexture remains inherently context-dependent. The prognostic impact of specific immune subsets may vary according to tumour type, disease stage, and therapeutic setting, and high immune cell density does not necessarily reflect effective antitumour immunity, particularly in the presence of immune exclusion or T-cell dysfunction.

Finally, potential publication bias towards positive findings and the absence of standardised clinical decision thresholds further complicate the translation of the IS into routine practice, highlighting the need for prospective validation and methodological harmonisation before widespread adoption (41–43).

## 3 Conclusions

Accumulating evidence across diverse solid tumours demonstrates that immune contexture, as captured by the IS and related composite immune classifiers, provides prognostic and predictive information that could complement TNM staging. The density, phenotype, and spatial organisation of tumour-infiltrating lymphocytes, particularly cytotoxic and memory T-cell subsets, emerge as central determinants of disease outcome and therapeutic benefit.

Importantly, the evolution of the IS from manual immunohistochemistry towards digital pathology, computational modelling, and non-invasive imaging surrogates marks a critical step towards clinical scalability. Integration with genomic alterations, systemic inflammatory indices, and radiologic features further enhances its discriminatory power, enabling refined risk stratification even in early-stage disease where treatment decisions remain controversial. Despite these advances, widespread clinical adoption requires methodological harmonisation, standardised scoring thresholds, and prospective validation in multicentre trials. Embedding the IS into treatment algorithms, particularly for guiding adjuvant therapy, immunotherapy selection, and surveillance intensity, represents a logical and achievable next step.

Remarkably, whilst these diverse approaches are often conceptually linked to the IS framework, they represent distinct biomarker classes with different biological assumptions, methodological requirements, and levels of clinical validation. Clear differentiation between these categories is essential to avoid overinterpretation and to guide their appropriate clinical application.

In summary, the IS and its modifications constitute a biologically grounded framework that bridges tumour immunology and clinical oncology. Its continued refinement and validation have the potential to modify immune profiling from a descriptive research tool into a cornerstone of precision cancer medicine.

## Glossary

ACT: adoptive T-cell therapy
APE: arterial-phase enhancement
BM: brain metastasis
BC: bladder cancer
dNLR: derived neutrophil-to-lymphocyte ratio
EGFR: epithelial growth factor receptor
GC: gastric cancer
HNSCC: head and neck squamous cell carcinoma
HCC: hepatocellular carcinoma
IM: invasive margin
IMPRS: Immune Prognostic Risk Score
IS: Immunoscore
MRI: magnetic resonance imaging
NK: natural killer
NSCLC: non-small-cell lung cancer
OSCC: oral squamous cell carcinoma
PIV: Pan-Immune Inflammation Value
RIS: Radiomic Immunoscore
SIRI: Systemic Inflammatory Response Index
TC: tumour core
TILs: tumour-infiltrating lymphocytes
TLS: tertiary lymphoid structure
TME: tumour microenvironment
TMS: tumour mutation score
